# Repatterning of the inflorescence meristem in *Gerbera hybrida* after wounding

**DOI:** 10.1007/s10265-021-01253-z

**Published:** 2021-02-04

**Authors:** Teng Zhang, Feng Wang, Paula Elomaa

**Affiliations:** Department of Agricultural Sciences, Viikki Plant Science Centre, University of Helsinki, P.O.Box 27, 00014 Helsinki, Finland

**Keywords:** Asteraceae, Auxin, Flower head, Laser ablation, Phyllotaxis, Wounding

## Abstract

The Asteraceae plant family is characterized by inflorescences, called flower heads or capitula that may combine hundreds of individual florets into a single flower-like structure. The florets are arranged in a regular phyllotactic pattern with Fibonacci numbers of left- and right-winding spirals. Such a pattern may be disrupted due to physical constraints or by wounding occurring during the early meristem development. Recovery from wounding re-establishes patterning although the mechanisms have remained elusive. In this study, we applied *Gerbera hybrida* as a model system and established methods to conduct wounding experiments either with syringe needles or using laser ablation combined with live imaging of head meristems. By revisiting the historical experiments in sunflower, we conducted wounding to transgenic auxin reporter lines of gerbera and followed the recovery of cellular growth and meristem patterning. We show that wounding disrupted the expression of the gerbera *CLAVATA3* (*GhCLV3*) gene that marks the undifferentiated meristematic region and led to *de novo* re-initiation of patterning at the wound margin. During the recovery growth, three to five layers of elongated cells showing periclinal cell division planes and lacking auxin signal were formed at the wound rim. DR5 auxin signal was shown to localize and form regularly spaced maxima in a distance from the wound rim. Consequently, spiral pattern of contact parastichies was re-established by stacking of new auxin maxima on top of the previous ones. The developed methods facilitate future studies on understanding the molecular mechanisms of *de novo* patterning of meristems.

**Supplementary Information:**

The online version contains supplementary material available at 10.1007/s10265-021-01253-z.

## Introduction

**Fig. 1:**
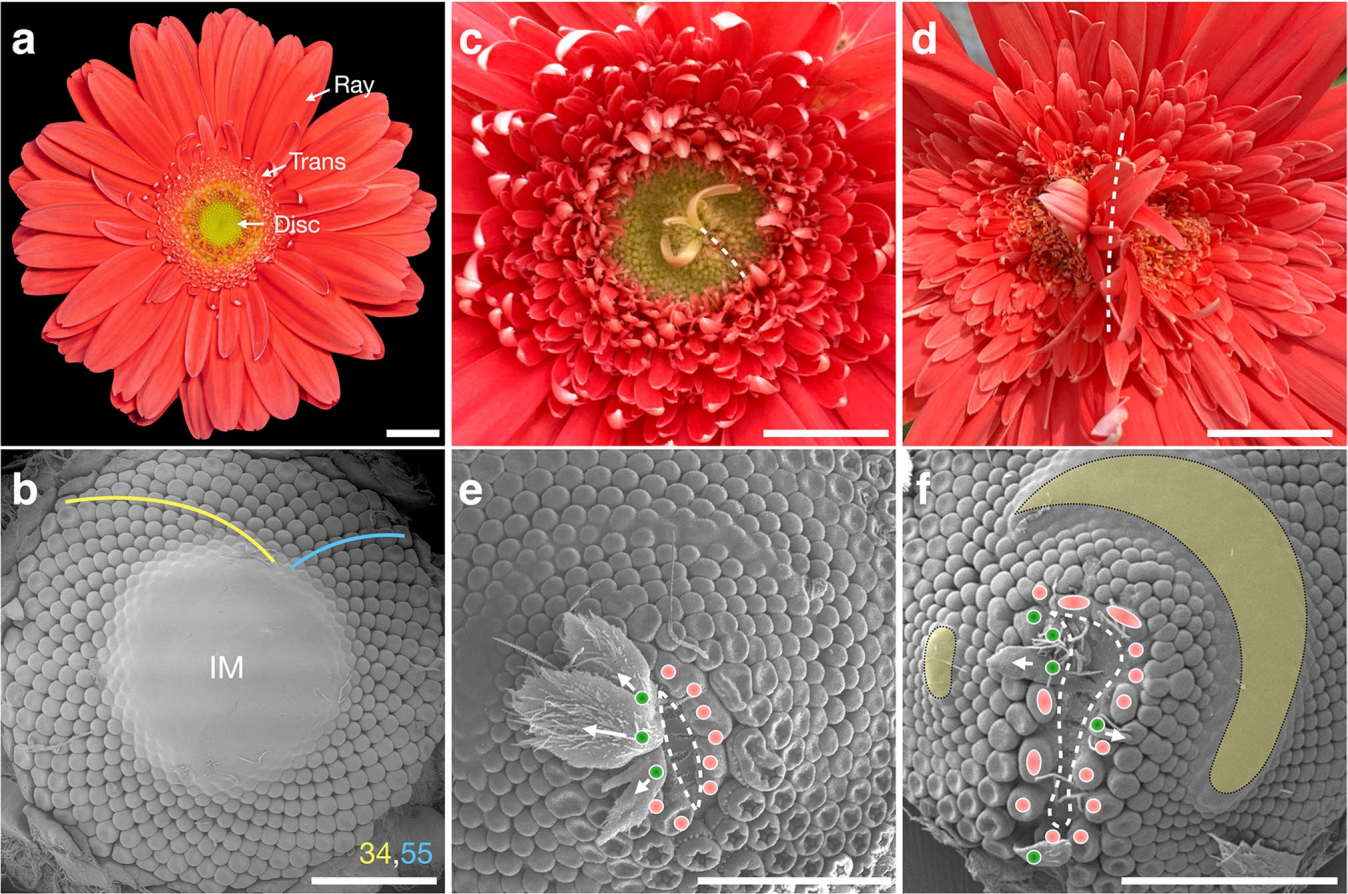
Wild type and naturally damaged flower heads of *Gerbera hybrida*. **a** Top view of a flower head of gerbera cv. Terra Regina comprising of three different types of florets; the marginal ray, intermediate trans and central disc florets. **b** SEM image of a gerbera head meristem at early stage. The individual floret primordia are arranged in 34 and 55 left and right turning spirals, respectively. At this stage, the center of the meristem (IM) is undifferentiated. **c**, **d** Top views of two naturally wounded flower heads. The wound sites are indicated by dashed lines. e, f SEM images of the two wounded head meristems corresponding to (**b**) and (**c**). Along the wound rim (marked by dashed lines), new involucral bract (green dots) or floret (red dots) primordia are formed. The bracts are orientated away from the wound rim (white arrows). The false-colored yellow regions in (f) indicate undifferentiated meristematic regions of the parent head. Scale bars: 1 cm (**a**, **c**, **d**), 1 mm (**b**, **e**, **f**)

Asteraceae, the sunflower family, is one of the largest families in the plant kingdom. The distinctive feature in species of this family are their unique flower-like inflorescences, known as flower heads or capitula (Elomaa et al. 2018). A flower head often combines up to hundreds of individual florets and bracts onto a single receptacle, while the overall structure resembles a single, solitary flower (Fig. 1a). Within a flower head, individual florets are arranged into highly regular left- and right-winding spirals, the number of which follows two consecutive numbers in the Fibonacci series (1, 1, 2, 3, 5, 8 ,13, 21…) (Fig. 1b). A pattern as such has attracted multidisciplinary researchers over centuries, and it is perceived as an iconic example to illustrate the geometric beauty in plants.

In plants, aerial organs are produced by a small group of cells in the shoot apical meristem (SAM). Within the dome-shaped SAM, such as in the model plant *Arabidopsis thaliana* L. Heynh., the stem cells are maintained in the central zone which generates daughter cells leading to their subsequent differentiation into new organs at the periphery of the meristem (Carles and Fletcher 2003). Compared to the relatively constant size of the Arabidopsis vegetative SAM, or the inflorescence meristem (IM) after floral induction, the IMs in Asteraceae show unique growth dynamics. They undergo significant expansion growth early in their development leading to physical size of approximately 10–20 times larger than those in Arabidopsis. For example, an early stage head meristem without any visible florets in sunflower or gerbera is 1–2 mm in diameter while the IM of Arabidopsis is 60–70 µm (Laufs et al. 1998; Palmer and Marc 1982; Teng Zhang, Mikolaj Cieslak, Paula Elomaa and Przemyslaw Prusinkiewicz, unpublished results). Recent data indicates that this dynamic growth mode of heads regulates their phyllotactic patterning (Teng Zhang, Mikolaj Cieslak, Paula Elomaa and Przemyslaw Prusinkiewicz, unpublished results).

Due to its large size and easy accessibility, the meristem of sunflower has historically been used as a model to study the effects of wounding, compression as well as chemical treatments on patterning of flower heads. The studies conducted mainly in 1980’s indicated that after making punctures or linear cuts (Palmer and Marc 1982) or cylindrical wounding (Hernandez and Palmer 1988) to the yet undifferentiated central region of a young sunflower head meristem, new involucral bracts, ray and disc florets, in this particular order, initiated from the wound rim. Wounding at late developmental stages did not induce bract or ray floret development indicating that the disc floret pattern was already established and could not be disrupted by wounding (Palmer and Marc 1982). Also in case of physical compression ectopic bracts formed in the centre of the flower head (Hernandez and Green 1993). The mechanisms for such effects have remained elusive. Re-patterning of primordia occurs fast (Palmer and Marc 1982; Palmer and Steer 1985) suggesting that for example chemical diffusion across the head is not a feasible explanation (Hernandez and Palmer 1988). Moreover, in case of cylindrical wounding, that created a fully separated, plug-like region at the meristem center, patterning continued along the expansion of the surface of this plug indicating that it is self-organizing. Yet, the parastichies in these secondary inflorescences were irregular and did not show Fibonacci numbers (Hernandez and Palmer 1988). Recently, Zoulias et al. (2019) showed that auxin provides a developmental cue for patterning by forming a temporal gradient that regulates involucral bract and floret identities in a concentration dependent manner. They also propose that wounding may physically block auxin flow, leading to accumulation of auxin in the rim of the wound.

In this study, our aim was to establish methods to conduct wounding experiments in *Gerbera hybrida* Hort. and to discover the effects on the growth and patterning of head meristems. We applied the previously made *DR5rev*::3xVenus-N7 auxin reporter lines of gerbera (Teng Zhang, Mikolaj Cieslak, Paula Elomaa and Przemyslaw Prusinkiewicz, unpublished results) that allowed us to visualize auxin patterning after the treatments. The classical wounding experiments of sunflower were revisited by applying an *in vitro* method for the growth of the wounded heads. Moreover, we optimized a targeted laser ablation method combined with live imaging of heads. Our data faithfully confirmed the previous results in sunflower indicating *de novo* establishment of patterning after wounding. We also showed that wounding disrupted *GhCLV3* expression that marks the undifferentiated meristem. DR5 signal was shown to localize in a distance from the wound margin and to initiate regularly spaced maxima indicating reprogramming of organ fates. The developed system provides a working tool for future studies to understand the instructive signals and molecular mechanisms for *de novo* patterning.

## Materials and methods

### Plant material

The wild type and transgenic plants of *Gerbera hybrida* cv. Terra Regina were grown under standard greenhouse conditions as previously described (Ruokolainen et al. 2010). For mechanical wounding and laser ablation, flower heads at early developmental stages (< 2 mm in diameters) were collected from two independent transgenic gerbera lines TR3 and TR7 (Teng Zhang, Mikolaj Cieslak, Paula Elomaa and Przemyslaw Prusinkiewicz, unpublished results) expressing the *DR5rev*::3xVENUS-N7 auxin reporter (Heisler et al. 2005). For live imaging, the wounded flower head specimens were surface sterilized using 5% Plant Preservative Mixture (PPM, Plant Cell Technology, USA), and were placed on gerbera multiplication media (Elomaa et al. 1993) supplemented with 1 mg l^− 1^ gibberellic acid (Duchefa, Biochemie B. V., Haarlem, The Netherlands), 0.1% PPM and 45 mg l^− 1^ sucrose.

### Mechanical wounding of the head meristem

The mechanical wounding of the growing head meristems was performed by modified syringe needles. Needles with variable inner diameters (0.3 mm, 0.45 mm and 0.8 mm; FINE-JECT®, Henke-Sass Walf, Tuttlingen, Germany) were selected. The tip of needles was firstly blunted by a knife blade, and their edges were then sharpened with a sharpening stone. For sample preparations, heads with sizes ranging from 1 to 3 mm in diameter were selected. At this stage, the IMs remain undifferentiated and large in size. Involucral bracts in these samples were removed to expose the meristem. Mechanical wounding was performed manually by gently pressing the needle on top of the dissected and sterilized head meristem explants under a stereomicroscope. In addition to cylindrical cuts, curved wounding was made by pressing one side of the needle on the meristem. The wounded head explants were cultured on Petri dishes on growing medium for 6 days, and further used for *in situ* hybridization and microscopic analyses.

### *In situ* hybridization of *GhCLV3*

The expression of a meristematic marker gene gerbera *CLAVATA3* (*GhCLV3*) in wounded head meristems was examined by *in situ* hybridization. The samples corresponding to distinct stages before and after wounding were fixed, sectioned and hybridized as described in Elomaa et al. (2003). A probe corresponding to the full length 327 bp coding region of *GhCLV3* was applied, and synthesized with primers containing a T7 overhang as described previously (Juntheikki-Palovaara et al. 2014). The following primer sequences were used: GhCLV3-F: AAAAAGCAGGCTCGATGGTTTTTTCACTCAGATATC, GhCLV3-R: AGAAAGCTGGGTTTAAGGAGTTCGGGGCTTTTTC, GhCLV3-F-SET7: CATAATACGACTCACTATAGGGATGGTTTTTTCACTCAGATATC, and GhCLV3-R-AST7: CATAATACGACTCACTATAGGGTTAAGGAGTTCGGGGCTTTTTC. Sections were visualized and imaged using a Leitz Laborlux S microscope equipped with a Leica DFC420 C digital camera.

### Laser confocal microscopy, image analysis and visualization

Head meristem samples of gerbera transgenic lines expressing the *DR5rev*::3xVENUS-N7 were examined by a Leica SP5 MP confocal microscope with an HCX APO L 20x/1.0W water dipping objective (Leica Mikrosysteme Vertrieb GmbH, Wetzlar, Germany). Fluorescence signals of VENUS and propidium iodide (PI) were simultaneously excited by an Argon laser at 514 nm, and their emissions were collected at 519–537 nm and 624–695 nm wavelengths, respectively. *Z*-stack scanning was made to all samples to allow 3D imaging. The analysis and visualization of the 3D image stacks from confocal microscope was performed by Fiji (Schindelin et al. 2012) and MorphoGraphX (de Reuille et al. 2015), according to the user manual.

### Laser ablation

For laser ablation of gerbera head meristems, the MaiTai IR laser coupled with the Leica SP5 MP confocal microscope was used. In order to obtain an overview of the meristem structure, each head sample was stained by PI and imaged before the ablation. The laser ablation was performed using the Leica LAS software for head meristems growing on Petri dishes. The multiphoton laser was set at a wavelength of 860 nm, and the trans, gain and offset values were set at 5% to achieve the smallest possible wounded area. A 20x water dipping objective was used and 64x zoom targeted to the designated cells, and ablation was made by a single pulse of capture with a shutter speed of 1400 Hz. For continuous ablations, multiple pulses were made to a single focusing plane to achieve a curve-shaped (approximate 20 pulses), or a cylindrical wound (approximate 100 pulses). The wounded head was imaged right after the ablation, and grown on medium for 4 days before imaging at a second time point.

### Scanning electron microscopy (SEM)

SEM was used to analyze the morphological details of flower heads in response to various types of wounding. For insect damaged heads, representative samples were observed and collected during routine sampling of wild-type gerbera in the greenhouse. Sample preparation was performed as previously described (Zhang et al. 2017). The samples were imaged by a Quanta 250 SEM (FEI Corp., USA) with accelerating voltage of 5.0 kV. The region of interest in the SEM images were highlighted by Adobe Photoshop CC.

## Results

### Naturally occurring wounding reinitiates patterning in flower heads

Wounding is known to initiate cellular dedifferentiation and subsequent regeneration of organs in flower heads (Hernandez and Palmer 1988; Palmer and Marc 1982). Similarly, boron deficiency resulted in surface splits and development of involucral bracts and ray florets in the centre of the sunflower receptacle (Blamey 1976; Hernández 2002). In gerbera, such abnormalities in flower heads can result from natural damage caused by insect bites or by tissue compression during cultivation under standard greenhouse conditions (Fig. 1c, d). Depending on the size, shape and place of the damage, they may either result in ectopic formation of involucral bracts or ray florets in the middle of the head (Fig. 1c), or in a more severe case, the head surface may completely split (Fig. 1d). In both cases the regular phyllotactic pattern is disrupted, as ectopic bracts and ray florets forming along the wound edges take the places normally occupied by disc florets.

To better understand the changes in morphology and patterning caused by such damage, we collected naturally wounded head samples of gerbera and observed them using SEM (Fig. 1e, f). Regardless of the shape and size of the cracked area, new primordia formed right at the edges of the wound. These primordia showed uniform organ polarity, with the abaxial side of bracts or ray florets pointing towards the wounded site. This is consistent with the previous studies on sunflower heads suggesting that the wound rim acts as an organizing center for formation of new primordia (Hernandez and Palmer 1988; Palmer and Marc 1982). We also noticed subtle differences in gerbera compared to earlier results reported in sunflower. Firstly, the identity of the primordia surrounding the wound margin in gerbera was not uniform. Based on their morphology observed by SEM, the emerging primordia were either involucral bracts or floret primordia (Fig. 1e, f). Secondly, some primordia were fused at the base resulting in non-uniform size of the primordia (Fig. 1f). These differences may result from the irregular contour of the rim caused by natural damage as compared to the uniform circular (Hernandez and Palmer 1988) or straight cuts (Hernandez 2002) conducted to sunflower heads. Nevertheless, after patterning of the first primordia at the rim, the following primordia were equalized in size and the spiral organization of primordia resumed as the pattern progressed away from the wound margin (Fig. 1e, f). At the same time, the natural pattern in the parent head progressed normally, until meeting the pattern front originating from the wound site. Both pattern fronts continued as long as the undifferentiated meristem space was available (marked in yellow in Fig. 1e, f).

### Growth and auxin responses at the site of mechanical wounding

**Fig. 2:**
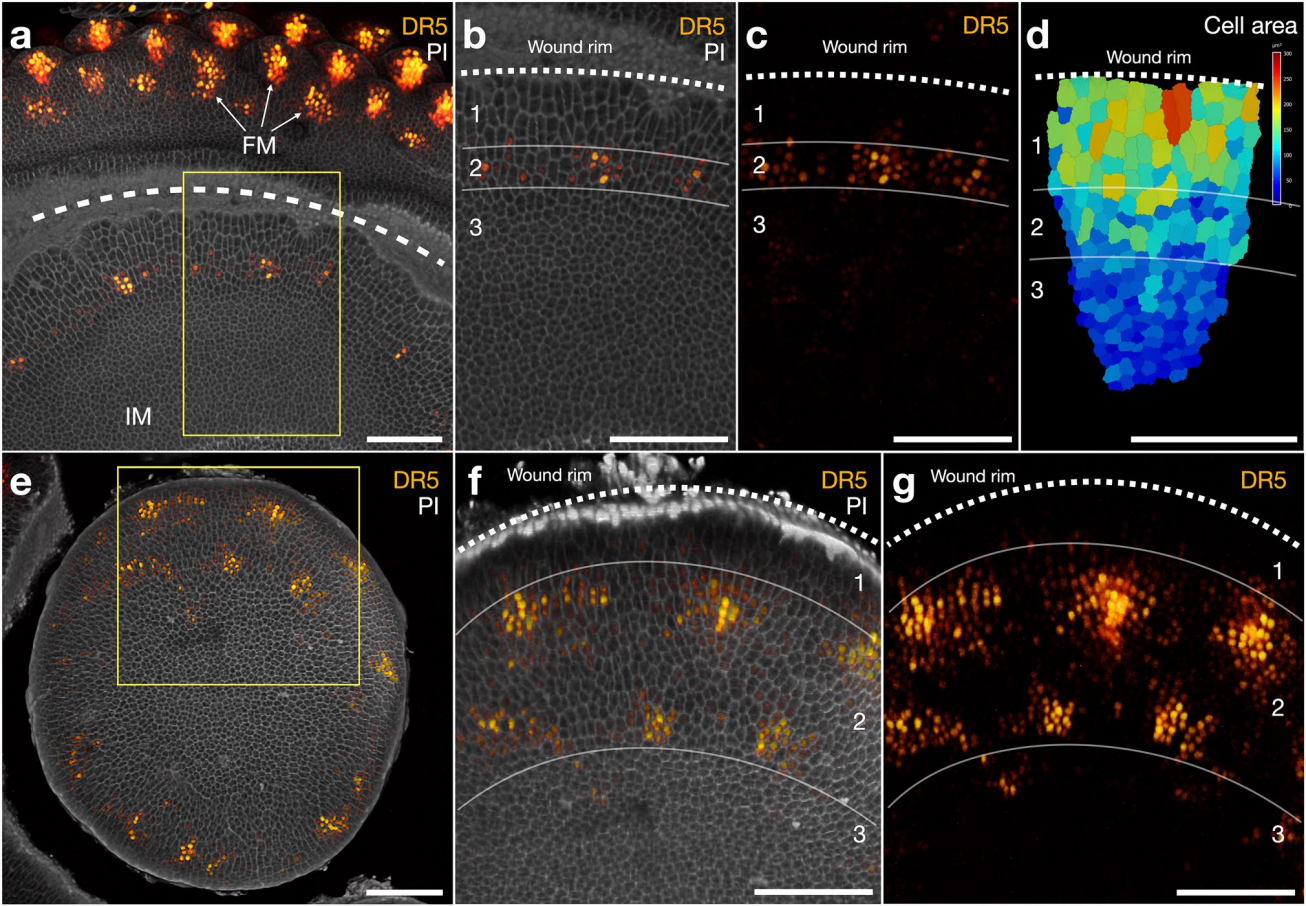
Effect of mechanical wounding to the growing head meristem in transgenic gerbera plants expressing the DR5 auxin reporter. **a** Top view of 3D reconstruction from *Z*-stack confocal microscopic images of a curved wound on a growing head meristem after 6 days culture on tissue culture media. The position of the wound is marked by the dashed white line over the region of dead cells. DR5 signal marks the position of incipient flower meristems (FM). Individual cells are stained using propidium iodide (PI). The region of already patterned FMs and the undifferentiated central inflorescence meristem (IM) are separated by the wound. **b**, **c** Close-up of the wound margin site as indicated by the inset in (**a**). DR5 signals localize in a distance of the wound rim (region 2) and starts to form regularly spaced maxima. **d** Measurement of cellular sizes (surface areas) in distinct regions shown in (**b**). The marginal cells next to the wound rim are larger in size and show significant elongation growth and periclinal division planes while the cells towards the center are small and more round-shaped. **e** Top view of 3D reconstruction from *Z*-stack confocal microscopic images of a growing head meristem after cylindrical wounding that separates a central plug from the parent head. **f**, **g** Zoomed in view of the head indicated by the inset in (**e**). DR5 signals indicate auxin patterning that is progressing towards the centre of the plug. Scale bars: 100 μm

To investigate the possible role of auxin at the wound site, we performed mechanical wounding to transgenic gerbera lines expressing the auxin reporter *DR5rev*::3xVENUS-N7 (Heisler et al. 2005) (Fig. 2). By utilizing modified syringe needles with different inner diameters, we were able to achieve either curved (Fig. 2a–d) or cylindrical wounds, creating a separated plug (Fig. 2e–g), to the yet undifferentiated growing meristems. By imaging the head meristems 6 days after the wounding, the wounded areas were found to be approximately 20–50 µm wide and 100–200 µm deep (Figs. 2a, e, S1), and thus comparable to those reported in sunflower treatments (Hernandez and Palmer 1988). We obtained confocal images from a total of seven wounded heads showing that wounding efficiently separated the treated area from the rest of the growing flower head (Fig. 2).

We analyzed the confocal images by focusing on cellular organization and DR5 expression at the wound site. The cells around the wound rim were found to be rearranged into three distinct regions. The first region, closest to the wound rim, is characterized by cells that show significant radial elongation and periclinal division planes in the direction parallel to the cut surface of the wound (Fig. 2b, region 1). This region comprises three to five layers of cells, which is consistent with the previous results in sunflower (Hernandez and Palmer 1988). Interestingly, the DR5 signals were completely absent in this region, indicating low auxin transcriptional output (Fig. 2c). Next to the elongated cells, a second region contains a ring of cells showing high expression of the DR5 reporter (Fig. 2b, c, region 2). DR5 signals in these cells started to localize into maxima, which formed approximately equidistant from each other (Fig. 2c). The adjacent auxin maxima, however, were not equal in size. This was seen in both the curved and the cylindrically wounded samples (Fig. 2c). Closest to the undifferentiated meristem center, cells in the third region remain undifferentiated and small in size (Fig. 2d, region 3). They also lack DR5 expression suggesting that these cells maintain similar identities (Fig. 2c). In heads with circular wounds, similar regional organization of cells were observed (Fig. 2e–g), except that after 6 days of recovery growth, the DR5 pattern had already progressed further towards the center of the isolated circle, and new maxima formed by stacking on top of the previously formed ones (Fig. 2g).

### ***GhCLV3*** expression is excluded from the wound margin

**Fig. 3:**
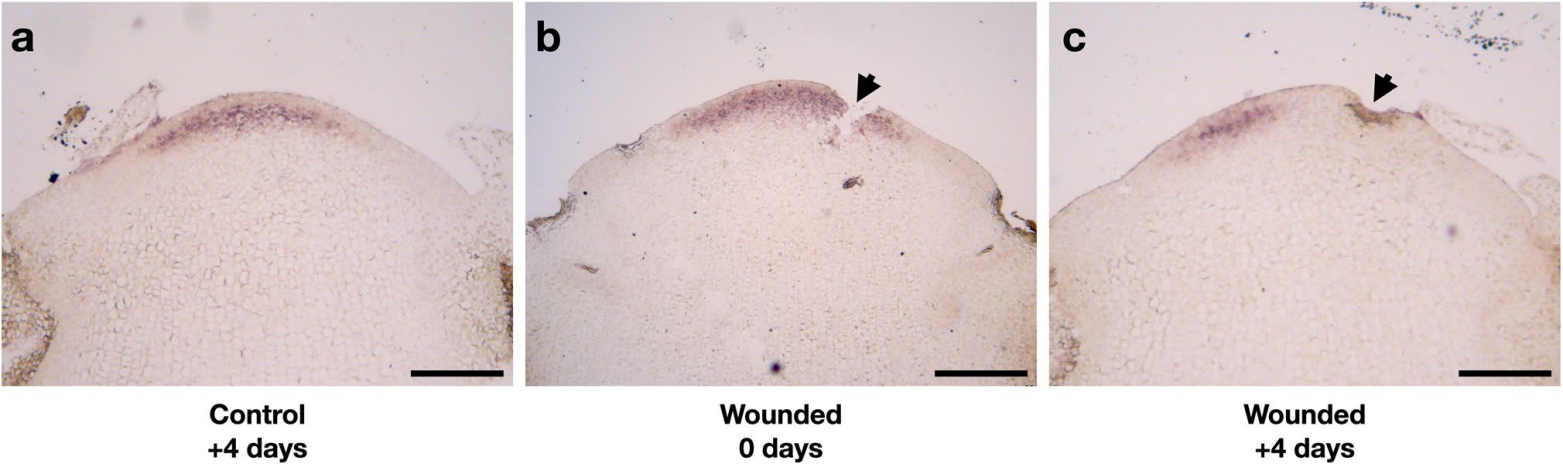
Gerbera *GhCLV3* expression shown by in situ hybridization in control and wounded head meristems. **a** *GhCLV3* expression domain in an intact head meristem grown on medium for 4 days. **b** *GhCLV3* expression immediately after the wounding. **c** *GhCLV3* expression in a wounded head meristem grown on medium for 4 days. Black arrowheads in (**b**, **c**) indicate the site of the wounding. Scale bars: 200 μm

The elongation of marginal cells closest to the wound rim, and the emergence of a ring of DR5 signals next to them indicate that their cellular fates might have changed during the recovery growth. Thereby, we examined the expression of *GhCLV3*, as a marker for undifferentiated central zone of the meristem (Teng Zhang, Mikolaj Cieslak, Paula Elomaa and Przemyslaw Prusinkiewicz, unpublished results), in the mechanically wounded heads (Fig. 3). In the non-wounded control sample, *GhCLV3* is expressed in the undifferentiated meristematic dome and maintains its expression after 4 days of culturing on medium (Fig. 3a). In the wounded sample, mechanical wounding was applied to the undifferentiated meristem. The wound on the meristem surface thus disrupted the area with high *GhCLV3* expression (Fig. 3b). After 4 days of recovery growth, *GhCLV3* expression was excluded from cells closest to the wound rim (Fig. 3c). The absence of *GhCLV3* in these cells suggests that their identity has been changed, and their fate no longer corresponds with those in the undifferentiated central domain.

### Laser ablation on epidermal layers recapitulates the effects of mechanical wounding

**Fig. 4:**
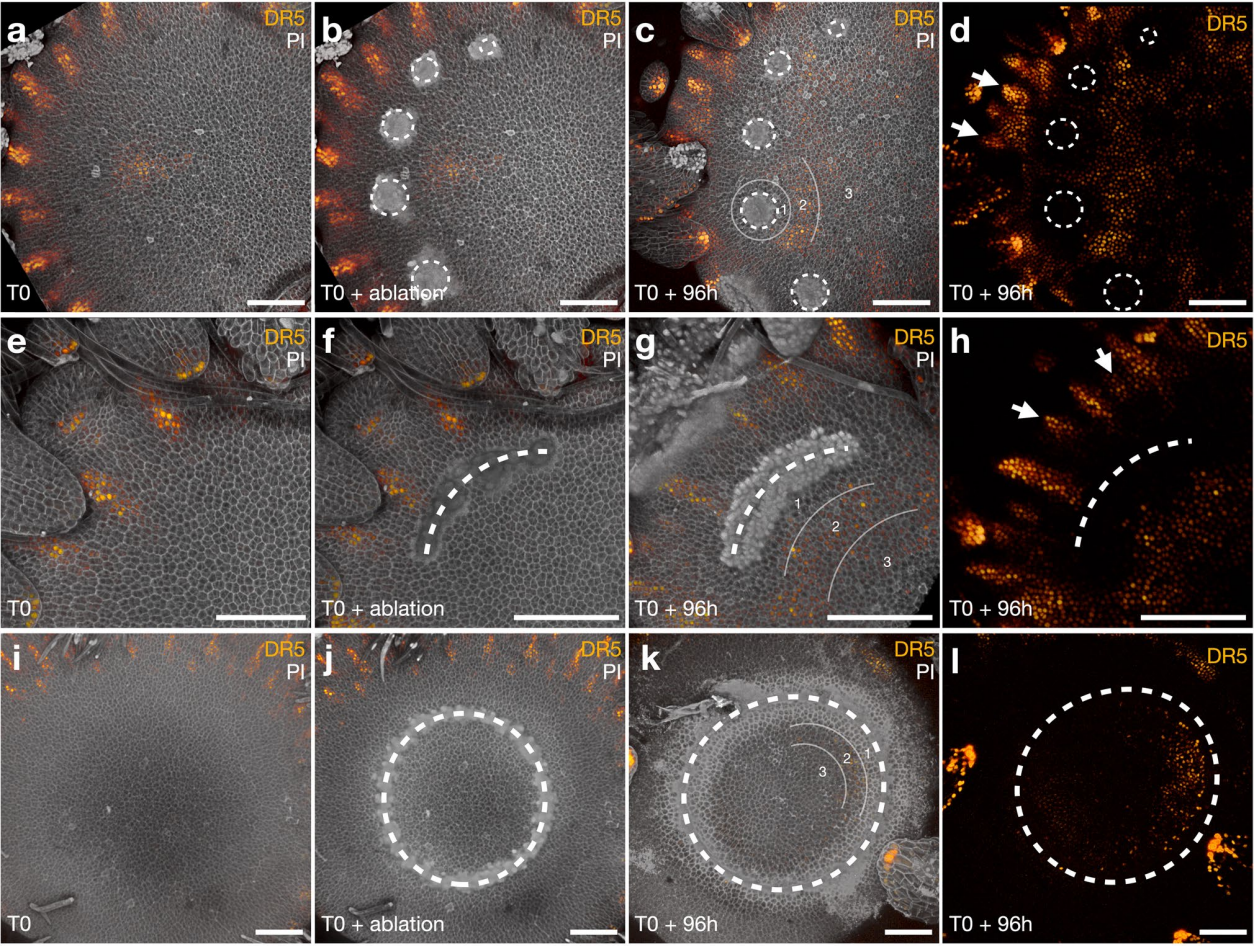
Effect of laser ablation to growing head meristem in transgenic gerbera expressing the DR5 auxin reporter. Top view of 3D reconstruction from *Z*-stack confocal microscopic images show live imaging of the same head meristem before (T0), just after ablation (T0 + ablation) and 4 days after ablation (T0 + 96h). **a**–**d** Five dot-shaped ablations (marked by dashed white circles) using different laser powers were produced. **e**–**h** A curved ablation (dashed white line) was achieved by lining multiple dot ablations. Note, patterning outside the wound area is progressing normally with new DR5 maxima initiating between the already existing maxima (white arrows in **h**). **i**–**l** A circular wound (dashed white circle) was achieved by lining multiple dot ablations. In all cases, three distinct regions near the wound site were observed. Auxin accumulation in region 2 was visible towards the centre of the meristem. Scale bars: 100 μm

In order to precisely control the size and dimensions of the wounded areas, we optimized a laser ablation protocol for gerbera head meristems by using a confocal microscope equipped with the multiphoton laser. PI staining was applied to each sample to locate the individual cells. During the ablation process the residual PI stains the nuclei of the dead cells, thus allowing us to visualize the ablated area immediately (Fig. 4). After optimizing the parameters, we were able to achieve a dot shaped ablation area of five cells wide and two cells deep, i.e. considerably smaller than those achieved by mechanical wounding (Fig. S1). To create differentially shaped wounds, consecutive point ablations were made. We were able to produce either point-shaped (Fig. 4b), curve-shaped (Fig. 4f) or cylindrical (Fig. 4j) wounds by laser ablation, and follow their effects after 4 days (96 h) of growth on the culture medium.

The head meristems with laser ablations showed similar effects on cellular growth and auxin response as the mechanically wounded heads. Regardless of the shape of the ablated area, the zonal organization of cells and DR5 expression were similar around the wound rim (Fig. 4c, g, k). This indicates that ablation of cells in the epidermal layer is sufficient to reproduce the responses observed in the mechanically wounded heads where the wound extends deeper into the tissue. At the immediate vicinity of the rim, the cells were elongated and devoid of auxin signal. Interestingly, we found that phyllotactic patterning in the non-ablated area was not affected by the wounding close by (Fig. 4d, h). New DR5 maxima emerged in designated places determined by the previously formed neighbors, emphasizing that local interaction of primordia, rather than signals across the entire head meristems determines phyllotactic patterning in heads. Towards the center of the head, new DR5 signals started to emerge and at the 96 h timepoint the very first auxin maxima just started to initiate (Fig. 4c, d, g, h, k, l).

## Discussion

In this study, we explored the effects of natural and mechanical wounding on repatterning of gerbera head meristems. We established wounding methods using syringe needles and optimized a laser-based ablation method. During the recent decades, developments of laser-based methods have increased the precision of wounding treatments, and have facilitated our understanding of regeneration processes in roots (van den Berg et al. 1995; Canher et al. 2020; Marhava et al. 2019; Marhavý et al. 2016; Matosevich et al. 2020) and SAM (Caggiano et al. 2017; Reinhardt et al. 2003, 2005). Although the gerbera heads were grown only for four to six days on tissue culture media, the method combined with live imaging provides a working tool to extend the experiments to obtain detailed molecular insights of wound responses and pattern re-initiation.

**Fig. 5:**
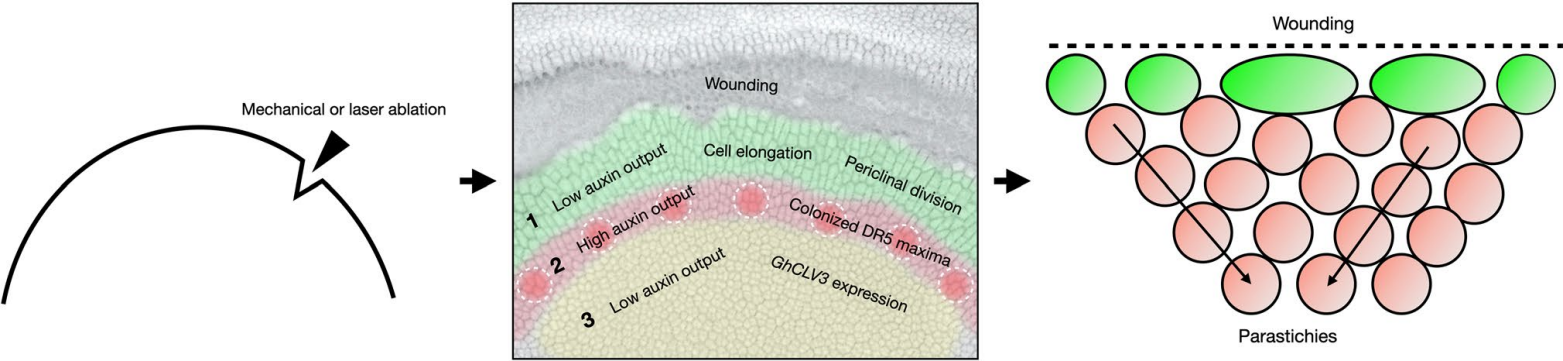
Schematic summary of gerbera head meristem responses to wounding. Both mechanical and laser-based wounding leads to similar responses in the re-establishment of cellular patterning. Region 1, closest to the wound rim, lacks auxin and is characterized by three to five layers of elongated cells with periclinal division planes. In region 2, DR5 signals localizes in regularly spaced maxima re-initiating phyllotactic patterning although in non-Fibonacci numbers. Region 3 retains high *GhCLV3* expression indicating yet undifferentiated nature of the cells. Regular contact parastichies are formed after the *de novo* pattern initiation

We revisited the classical wounding experiments conducted in sunflower, with the exception that the wounded gerbera heads were grown on tissue culture medium. Our observations confirmed the earlier results (Hernandez and Palmer 1988; Palmer and Marc 1982) indicating that mechanical wounding, and also naturally existing cracks on the head meristem surface, induce cell divisions and re-initiate organ patterning at the wound rim (summarized in Fig. 5). Similar response was observed when we applied laser ablation to the epidermal cells of the meristem, regardless of the shape of the ablated area. The first phases of cellular patterning in gerbera and sunflower seem to be strikingly conserved. In both systems, three to five layers of elongated cells are formed in the vicinity of the wound margin showing periclinal division planes (Hernandez and Palmer 1988). These rapid cell divisions seem to be a prerequisite for the regeneration process (Hernandez and Palmer 1988).

Using transgenic gerbera plants expressing the DR5 reporter, we were able to monitor auxin responses in the wounded meristems. Wounding separates the tissue from the rest of the flower head, and the auxin pattern was found to re-initiate from a distance of the rim while the cells just around the wound site were devoid of auxin signal. In case of cylindrical wounding (conducted near the margin of the head), the pattern progressed centripetally while from the central wounds or splits in naturally wounded heads (Fig. 1), the new primordia were oriented in both sides around the wound margin, and patterning progressed also in reverse direction, centrifugally regarding the parental head. The SEM figures from naturally wounded heads indicate that the primordia emerging from the wound site form regular contact parastichies. The number of parastichies does not necessarily follow Fibonacci numbers. The same effect was found earlier in sunflower and it was suggested that the primordia numbers most likely correspond to the wound size as linear cuts produced more bracts than punctured ones (Hernandez and Palmer 1988; Palmer and Marc 1982). The phyllotactic model in gerbera shows that patterning is a concurrent process where multiple auxin maxima emerge in bursts, and progress through Fibonacci numbers (Teng Zhang, Mikolaj Cieslak, Paula Elomaa and Przemyslaw Prusinkiewicz, unpublished results). In contrast, in a wound rim, a random number of auxin maxima is formed depending on the size of wound rim. The laser-ablated heads were grown for 4 days on the culture medium, and during this time period, only the very first auxin maxima started to initiate. Nevertheless, our results confirm the sunflower results suggesting that phyllotactic patterning is re-established *de novo* at both natural and mechanically induced wound rim, and does not need any external signal for example from leaves. Furthermore, as primordia may also form centrifugally from the wound site, patterning is not related to the radial symmetry of the head. We cannot exclude the involvement of signals from the underlying receptacle, possibly delivered through the vasculature. Still, the earlier sunflower studies argue against this hypothesis as the wounded plugs removed from the parent receptacle still re-established patterning (Hernandez and Palmer 1988).

The identity of organs formed after wounding follows a strict order. In case of gerbera, involucral bracts or ray florets first occupy the wound rim followed by disc florets. Hernandez and Palmer (1988) discuss that organ identity reflects their relative position with respect to the rim (i.e. those closest to the rim gain bract identity while those further away floret identity), or alternatively the first formed primordia may control the fate of later emerging ones. Zoulias et al. (2019) showed that an endogenous auxin gradient in flower heads defines the identity of involucral bracts and florets in a concentration dependent manner. Our data indicates high auxin response in a short distance from the wound margin and initiation of regularly spaced maxima that mark the positions of the future organs. Thereby, the identity of organs might be determined by the auxin level of the individual maximum. The cause for an auxin gradient proposed by Zoulias et al. (2019) may either result from directional auxin transport, or localized auxin biosynthesis near the wounded region. Future studies should be targeted to monitor auxin flow in *PIN1* marker lines and to quantify auxin levels with the ratiometric R2D2 reporter (Liao et al. 2015) over a longer period of patterning. At a genetic level, LEAFY (LFY) and CYCLOIDEA-like (CYC) TCP transcription factors are required to define the identity of ray florets in diverse Asteraceae species (Elomaa et al. 2018). In *Matricaria inodora* L. Fl. Suec., ed. 2 (Linnaeus) 297 (1755), high auxin was suggested to suppress *MiLFY* expression in the head margin to allow involucral bract development while auxin was shown to activate both *MiLFY* and a CYC-like *MiRAY2* gene expression in incipient ray floret primordia (Zoulias et al. 2019). It would be interesting to clarify whether this model applies to wounded gerbera heads by exploring the expression domains of *LFY* and ray specific *CYC*-like genes in relation to the observed auxin pattern.

Our results highlight conservation of wound responses in apical meristems across different species, regardless of the original size and shape of the meristem. In this study, the wounds created by mechanical or laser ablation were made to the undifferentiated central region marked by *GhCLV3* expression. After wounding, the observed zonal organization of cellular growth and auxin patterns indicate that the cell fates along the wound rim were reprogrammed. During a short period of recovery growth, *GhCLV3* expression disappeared exclusively from the cells near the wound region, at a distance comparable to cells exclusive of *WUSCHEL* expression in the wounded SAMs of tomato (Reinhardt et al. 2003). Moreover, periclinal divisions and radial elongation was found in cells in the immediate vicinity of the wound rim suggesting *de novo* re-establishment of a peripheral zone. DR5 signals instead were found in a few cells away from the wound rim in gerbera, and then localizing into maxima to mark the positions of future organs. Previous studies in Arabidopsis showed that laser-ablation in the SAM leads to dynamic reorientation of microtubule arrays circumferentially around the wound (Hamant et al. 2008). In addition, microtubule orientation is highly correlating with localization of the auxin efflux carrier PIN1 that becomes localized towards the anticlinal cell wall in parallel to microtubules in cells farthest away from the wound rim (Heisler et al. 2010). Heisler et al. (2010) propose that the influence on cell polarity including both microtubule reorientation and PIN1 localization may result as a response to upstream mechanical signals due to ablation. In Arabidopsis, regeneration of root meristem after wounding has recently shown to involve a combination of auxin signaling, polarized transport and highly precise activation of local auxin biosynthesis (Canher et al. 2020; Matosevich et al. 2020). Future studies should clarify whether the source of auxin in the wounded gerbera IM derives from PIN-mediated polar transport or from local biosynthesis during re-patterning.

Recent studies have also indicated a role for HD-ZIPIII and KANADI1 in the periphery of Arabidopsis SAM in defining the positions of new organs and pre-patterning of cell type boundaries affecting their subsequent morphogenesis (Caggiano et al. 2017). These genes were also shown to play a major role in reorganization of tissues after wounding. By analyzing the ratiometric auxin reporter R2D2, the authors found that low auxin levels around the wound coincided with upregulation of *KANADI1* that defines the peripheral cell fate (Caggioano et al. 2017). This pattern is consistent with the observed adaxial/abaxial orientation of emerging bracts around the wound rim in gerbera, and urges for further functional studies to clarify the possible involvement of *KANADI1* in specifying the margin of the head meristem and its role during re-patterning.

Head meristem, with its large size and unique spiral phyllotaxis, serves as an intriguing model to study cellular patterning and organ fate regulation. Laser capture microdissection of distinct cell layers at the wound margin combined with transcriptomics analysis would give insights into the molecular level changes during re-patterning. Moreover, the developed methods provide tools to disrupt emergence of single primordia putatively affecting local interactions between the neighboring primordia, and more generally the spiral phyllotactic patterning of heads.

## Supplementary Information

Below is the link to the electronic supplementary material.

Supplementary file1 Figure S1. Comparison of the size of physical wounding by needles and laser ablation. a-b Optical cross-sections of gerbera head meristems wounded by needle tips. (a) and (b) correspond to Fig 2a and Fig 2e, respectively. c-g Optical cross-sections of a gerbera head meristem with different laser power outputs (sample corresponding to Fig 4b). Note the laser power that generates the smallest ablated area (g) was used for subsequent ablation experiments. Scale bars: 100 μm (PDF 345 KB)
