# Supplementary material for: Repatterning of the inflorescence meristem in Gerbera hybrida after wounding
Source: J Plant Res. 2021 Feb 4;134(3):431–40. doi: 10.1007/s10265-021-01253-z (PMC8106577; doi:10.1007/s10265-021-01253-z)
Supplement: Supplementary file 1 — Supplementary file1 Figure S1. Comparison of the size of physical wounding by needles and laser ablation. a-b Optical cross-sections of gerbera head meristems wounded by needle tips. (a) and (b) correspond to Fig 2a and Fig 2e, respectively. c-g Optical cross-sections of a gerbera head meristem with different laser power outputs (sample corresponding to Fig 4b). Note the laser power that generates the smallest ablated area (g) was used for subsequent ablation experiments. Scale bars: 100 μm (PDF 345 KB) [file 10265_2021_1253_MOESM1_ESM.pdf]

Supplemental material

Journal of Plant Research

Repatterning of the inflorescence meristem in *Gerbera hybrida* after wounding

Teng Zhang, Feng Wang, Paula Elomaa

Department of Agricultural Sciences, Viikki Plant Science Centre, University of Helsinki, Finland

Corresponding author: paula.elomaa@helsinki.fi

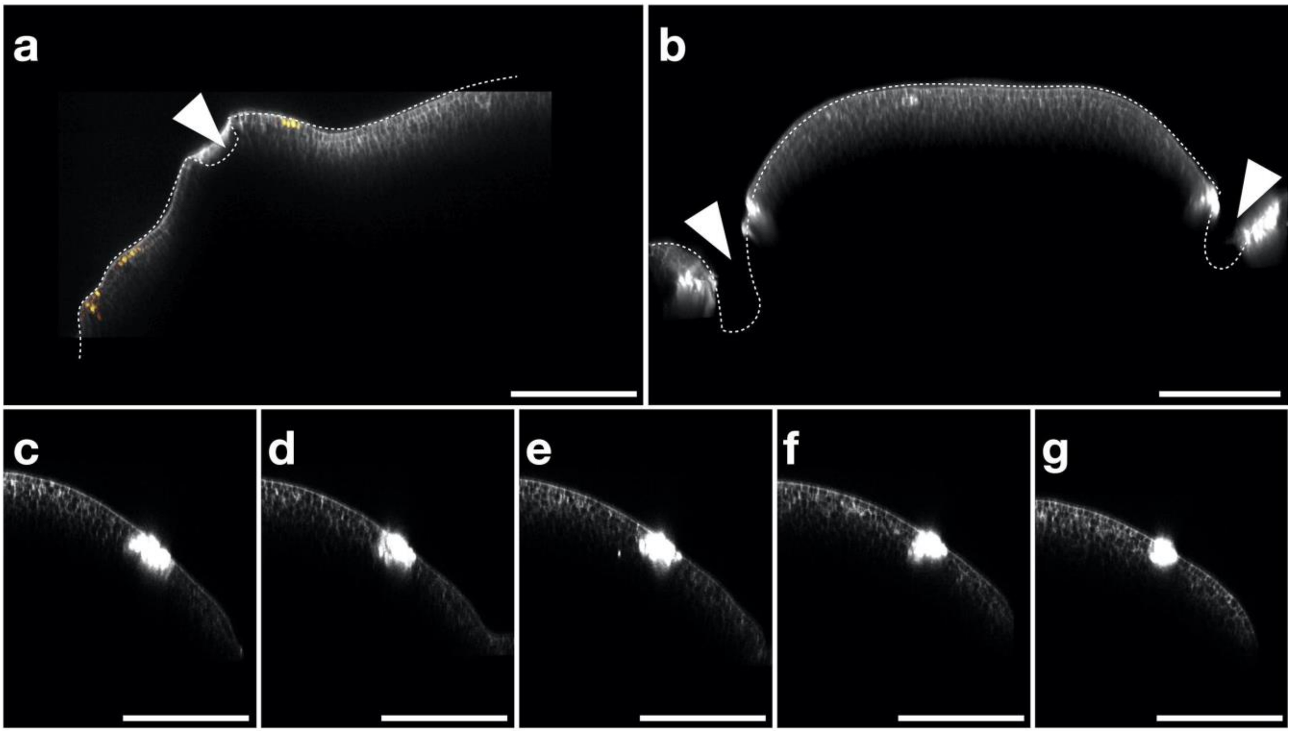

Figure S1. Comparison of the size of physical wounding by needles and laser ablation.
(**a-b**) Optical cross-sections of gerbera head meristems wounded by needle tips. (a) and (b) correspond to Fig 2a and Fig 2e, respectively.
(**c-g**) Optical cross-sections of a gerbera head meristem with different laser power outputs (sample corresponding to Fig 4b). Note the laser power that generates the smallest ablated area (g) was used for subsequent ablation experiments.
Scale bars: 100 µm.
